# Unstable Transcripts in *Arabidopsis* Allotetraploids Are Associated with Nonadditive Gene Expression in Response to Abiotic and Biotic Stresses

**DOI:** 10.1371/journal.pone.0024251

**Published:** 2011-08-29

**Authors:** Eun-Deok Kim, Z. Jeffery Chen

**Affiliations:** Section of Molecular Cell and Developmental Biology, Center for Computational Biology and Bioinformatics, and Institute for Cellular and Molecular Biology, The University of Texas at Austin, Austin, Texas, United States of America; University of Massachusetts Amherst, United States of America

## Abstract

Genome-wide analysis has documented differential gene expression between closely related species in plants and animals and nonadditive gene expression in hybrids and allopolyploids compared to the parents. In *Arabidopsis*, 15–43% of genes are expressed differently between the related species, *Arabidopsis thaliana* and *Arabidopsis arenosa*, the majority of which are nonadditively expressed (differently from mid-parent value) in allotetraploids. Nonadditive gene expression can be caused by transcriptional regulation through chromatin modifications, but the role of posttranscriptional regulation in nonadditive gene expression is largely unknown. Here we reported genome-wide analysis of mRNA decay in resynthesized *Arabidopsis* allotetraploids. Among ∼26,000 annotated genes, over 1% of gene transcripts showed rapid decay with an estimated half-life of less than 60 minutes, and they are called allotetraploid genes with unstable transcripts (*AlloGUTs*). Remarkably, 30% of *alloGUTs* matched the nonadditively expressed genes, and their expression levels were negatively correlated with the decay rate. Compared to all genes, these nonadditively expressed *alloGUTs* were overrepresented 2-6-fold in the Gene Ontology (GOSlim) classifications in response to abiotic and biotic stresses, signal transduction, and transcription. Interestingly, the *AlloGUTs* include transcription factor genes that are highly inducible under stress conditions and circadian clock regulators that regulate growth in *A. thaliana*. These data suggest a role of mRNA stability in homoeologous gene expression in *Arabidopsis* allopolyploids. The enrichment of nonadditively expressed genes in stress-related pathways were commonly observed in *Arabidopsis* and other allopolyploids such as wheat and cotton, which may suggest a role for stress-mediated growth vigor in hybrids and allopolyploids.

## Introduction

Polyploidy (whole genome duplication) is common in many plants and some animals [1], [2], [3]. The polyploid plants may represent over 70% of angiosperms [4], [5], and >75% of which are allopolyploids [1], [6], [7]. Most important crops have a polyploidy origin. Wheat, cotton and canola are allopolyploid that consist of two or more divergent genomes [3], and some plants and animals exist as interspecific hybrids [8], [9]. The common occurrence of plant polyploids indicates an advantage of being allopolyploids in response to selection and adaptation [6], [10], [11], [12]. Stable allopolyploids with meiotic transmission of homoeologous genomes lead to permanent fixation of heteozygosity and hybrid vigor.

Stable *Arabidopsis* allotetraploids (*Arabidopsis suecica*-like) are resynthesized by crossing tetraploid *Arabidopsis thaliana* with *Arabidopsis arenosa* [13]. The resynthesized *A. suecica* allotetraploids show morphological and physiological differences from their progenitors [13], [14], [15]. Microarray analysis of gene expression indicated that 5–38% of the genes are nonadditively expressed (different from mid-parent value, MPV) in resynthesized allotetraploids [14]. Nonadditive expression suggests activation or repression of a gene through genetic and epigenetic mechanisms [16], [17], [18]. Gene activation or silencing in allotetraploids can be caused by transcriptional regulation through changes in chromatin structure. However, many expression assays including microarrays and RNA sequencing measure the steady levels of mRNA transcripts. It is unknown whether mRNA stability affects nonadditive expression of genes between allotetraploids and their progenitors, *A. thaliana* and *A. arenosa*.

Posttranscriptional regulation of mRNA plays an important role in gene expression through modulation of mRNA stability and turnover. For example, *c-myc* and *c-fos* transcripts in mammalian cells and mating-type transcripts in yeast are unstable [19], [20], [21]. The decay rate of mRNA is correlated with biological functions of the genes and the length of cell cycles in mammals and yeast [22], [23], [24]. There is a role for mRNA stability in shaping the kinetics of the transcriptome in response to environmental changes in yeast and mammals [25], [26]. Approximately 50% of stress responsive genes in human cells are controlled at the level of mRNA decay [27]. In yeast, mRNA turnover is a major mediator in the response to heat shock, nutrition deprivation, oxidative stress, and osmotic stress [22], [28], [29].

Genome-wide analysis of mRNA decay was studied in *Arabidopsis* [30], [31]. It is shown approximately 1% of *Arabidopsis thaliana* genes with unstable transcripts (*AtGUTs*) [30]. These *AtGUTs* include the genes that are induced by mechanical stimulation and circadian rhythms. Moreover, transcripts from intronless genes and microRNA target genes generally have short half-lives [31]. Unstable transcripts are also identified in photo-labile phytochrome genes [32] and auxin inducible genes [33], [34], which facilitates rapid turnover of mRNA, leading to tight and effective control of gene expression [35]. To test a role of mRNA stability in nonadditive gene expression in allotetraploids, we examined mRNA turnover rates in resynthesized *A. suecica* allotetraploids relative to the mid-parent value using oligo-gene microarrays [14]. The candidates of allotetraploid genes with unstable transcripts (*AlloGUTs*) were compared with the genes that are nonadditively expressed, as identified in a previous study [14]. We further analyzed Gene Ontology (GOSlim) classifications and compared *alloGUTs* with stress responsive genes in published microarray datasets. Finally, we validated expression for a subset of genes using qRT-PCR analysis. These comprehensive analyses revealed mRNA instability of the genes related to stresses and clock-mediated pathways, suggesting a role for rapid mRNA decay in regulating stress and circadian responses to growth vigor in allopolyploids.

## Results

### Genome-wide analysis of unstable transcripts in allotetraploids using oligo-gene microarrays

**Figure 1:**
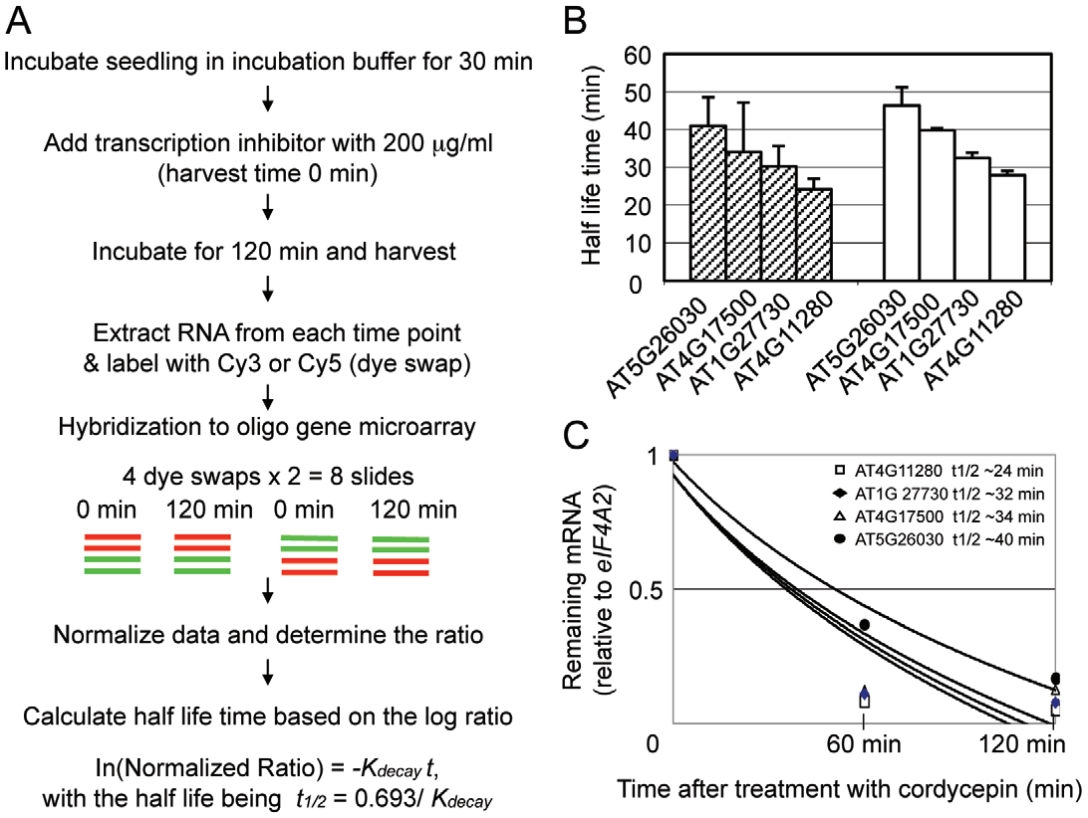
Identification of unstable transcripts with a half-life time of less than 60 min in *Arabidopsis* allotetraploids and validation of microarray data. (**A**) Experimental procedure for monitoring mRNA decay rates in *Arabidopsis* allotetraploids using spotted 70-mer oligo-gene microarrays. (**B**) qRT-PCR analysis (open bar) of half-life time of four genes with unstable transcripts identified by microarrays (hatched bar). (**C**) Quantification of mRNA decay rates of four genes and estimation of their half-life time.

Spotted oligo-gene microarrays were used to evaluate mRNA turnover in allotetraploids relative to the MPV (Figure 1A). The 70-mer oligos were designed from annotated genes and could cross-hybridize with *A. thaliana* and *A. arenosa* genes. This may underestimate changes in allotetraploids in which transcripts from one homoeologous locus display rapid decay, whereas the transcripts from the other locus are relatively stable. After data normalization and statistical tests (see *Methods*), the qualified values with reproducibly normalized intensity ratios of ≥2 were used for comparative analysis of gene expression. Approximately ∼1% (208) of genes in the *Arabidopsis* allotetraploid (Allo733) were identified as unstable transcripts with a half-life time of less than 60 min using the same criteria as previously defined for unstable transcripts [30]. These are hereafter referred to as allotetraploid genes with unstable transcripts (*AlloGUTs*) (Table S1). The percentage of *AlloGUTs* identified in allotetraploids is consistent with ∼1% of *AtGUTs* identified in *A. thaliana* using cDNA microarrays [30]. To confirm the microarray data, a subset of 10 genes was validated using quantitative RT-PCR (qRT-PCR) analysis, and expression patterns of four *AlloGUTs* were determined (Figure 1B, C). The four genes are: At5g26030 (*ATFC1*) encoding ferrochelatase 1, At4g17500 (*ERF1*) encoding ethylene responsive element binding factor 1, At1g27730 (*ZAT10*) encoding salt tolerance zinc finger, and At4g11280 (*ACS6*) encoding 1-aminocyclopropane-1-carboxylic acid (ACC) synthase 6. In qRT-PCR analysis, the gene encoding translation initiation factor 4A2 (*eIF4A2*, At1g54270) was used as a control because its transcripts were reasonably stable and did not depredate during the time course of chemical treatments [30]. The estimated turnover rate was 24–40 minutes by qRT-PCR (Figure 1C), which was comparable to 28–47 minutes estimated from microarray data (Figure 1B).

**Figure 2:**
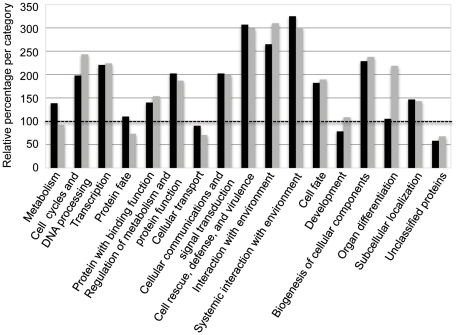
Gene Ontology (GOslim) classification of allotetraploid genes with unstable transcripts (*AlloGUTs*, black bar) relative to *Arabidopsis thaliana* genes with unstable transcripts (*AtGUTs*, gray bar). The two unstable gene sets were analyzed using TAIR release 9.0 and the PEDANT analysis system (http://mips.gsf.de/proj/thal/db/index.html). The proportion of the *GUTs* in each GOslim group was divided by the proportion of all genes in the genome (100%, dashed line).

A total of ∼208 *AlloGUTs* and ∼95 *AtGUTs* were classified into 20 GOSlim groups (Figure 2) using TAIR release 9 (November 2009) and PEDENT (http://mips.gsf.de/proj/thal/db/index.html). The proteins encoded by *AlloGUTs* were distributed among all GOSlim biological classifications. Compared with the proportion of the whole-genome annotated genes, *AlloGUTs* were overrepresented by a factor of two in the groups of cell cycle and DNA processing, transcription, regulation of metabolism and protein, and cellular communication and biogenesis of cellular component (Figure 2). In particular, the genes with unstable transcripts were enriched in four groups, namely, cell rescue, defense and virulence, interaction with environment, and systemic interaction with environment. They had significantly (2.6–3.2-fold) higher proportions of *GUTs* than that of all genes (dashed lines) [30] (Figure 2).

### Rapid mRNA turnover and nonadditive gene expression in allotetraploids

**Figure 3:**
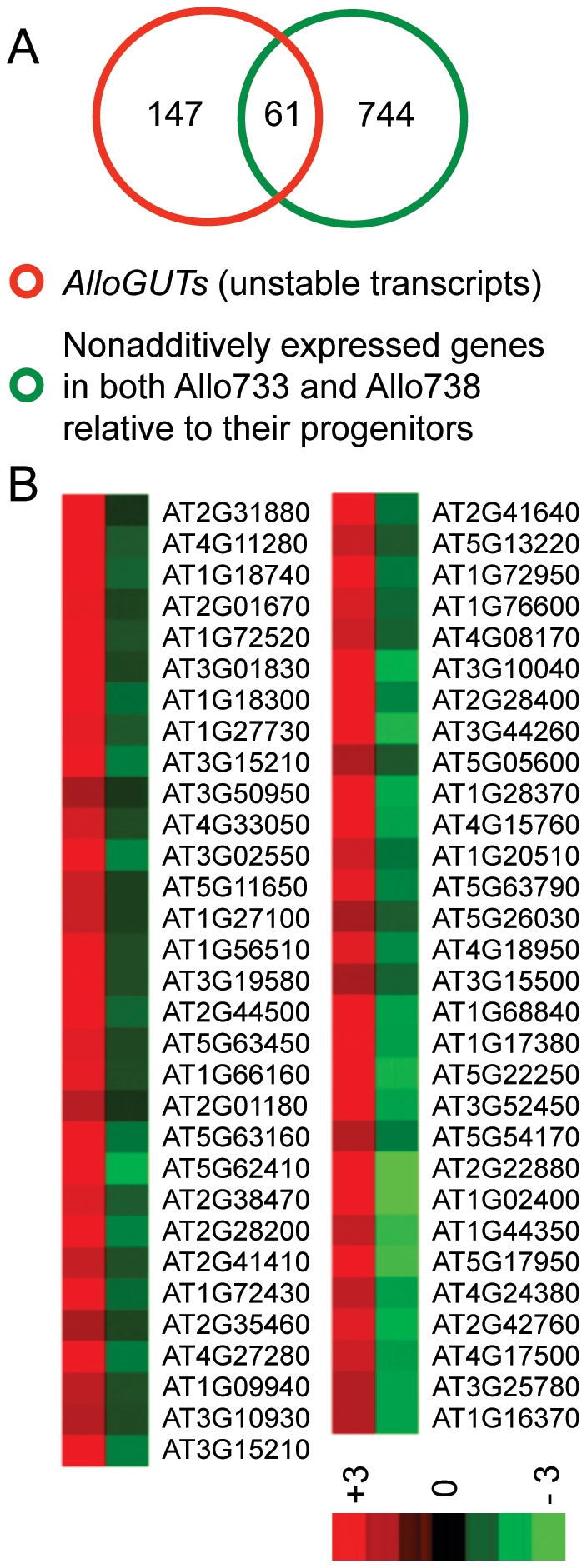
A relationship between *AlloGUTs* and nonadditively expressed genes in allotetraploids (Allo733 and Allo738). (**A**) Venn diagrams showing overlapping genes between *AlloGUTs* with half-life time less than 60 min and the common set of nonadditively expressed genes in both Allo733 and Allo738. (**B**) Heat map constructed from mRNA decay rates of *AlloGUTs* (first column, mainly red) and expression levels of nonadditively expressed genes (second column, mainly green). The bar shows relative levels of log2-fold changes (from −3 to +3).

Unstable transcripts are associated with mRNA stability and possibly steady levels of transcripts in allotetraploids. To test this, we compared the gene list of nonadditive expression identified in a previous study [14] with that of *AtGUTs* in *A. thaliana* diploid [30] and *AlloGUTs* in this study. Sixty-one of 208 (∼30%) *AlloGUTs* matched the nonadditively expressed genes in both allotetraploids (Allo733 and Allo738) (Table 1, Figure 3A). The correlation was statistically significant (degree of freedom  = 1, Chi-square  = 460.945, and p≤0.01) (Table S2). Hierarchical cluster analysis suggested a strong negative correlation between the expression levels of nonadditively expressed genes and mRNA decay rates of *AlloGUTs* (Figure 3B). The data suggest that these nonadditive expressions of the genes in the allotetraploids tested are related to rapid mRNA turnover. We also examined an enrichment of these genes in biological processes compared with the whole genome annotation. As in the nonadditively expressed genes [14], [36], the nonadditively expressed *AlloGUTs* were enriched 2–8 fold in GOSlim groups of stress response pathways, followed by response to abiotic or biotic stimulus, signal transduction, and transcription (Figure S1).

**Table 1:** List of nonadditively expressed *AlloGUTs*.

Locus	Description	t1/2(min)
AT5G62410	SMC2 (STRUCTURAL MAINTENANCE OF CHROMOSOMES 2)	16.86
AT3G44260	CCR4-NOT transcription complex protein, putative	18.81
AT3G02550	LBD41 (LOB DOMAIN-CONTAINING PROTEIN 41)	19.45
AT3G10040	transcription factor	20.54
AT3G15210	ATERF-4 (ETHYLENE RESPONSIVE ELEMENT BINDING FACTOR 4)	21.72
AT1G18740	similar to unknown protein	21.87
AT2G28200	nucleic acid binding/transcription factor/zinc ion binding	23.28
AT2G44500	similar to unknown protein	23.38
AT1G28370	ATERF11/ERF11 (ERF domain protein 11)	24.06
AT4G11280	ACS6 (1-AMINOCYCLOPROPANE-1-CARBOXYLIC ACID (ACC) SYNTHASE 6)	24.15
AT4G15760	DL3920C, FCAALL.376, MO1, MONOOXYGENASE 1	24.71
AT5G63160	BTB and TAZ domain protein	25.00
AT2G22880	VQ motif-containing protein	25.08
AT2G31880	leucine-rich repeat transmembrane protein kinase, putative	25.08
AT1G02400	ATGA2OX6/DTA1 (GIBBERELLIN 2-OXIDASE 6)	25.14
AT4G27280	calcium-binding EF hand family protein	25.36
AT1G72520	lipoxygenase, putative	25.51
AT1G18300	ARABIDOPSIS THALIANA NUDIX HYDROLASE HOMOLOG 4, ATNUDT4,	25.56
AT5G22250	CCR4-NOT transcription complex protein, putative	25.70
AT3G15210	ATERF-4(ETHYLENE RESPONSIVE ELEMENT BINDING FACTOR 4)	26.04
AT5G17950	unknown protein	26.05
AT3G01830	calmodulin-related protein, putative	26.98
AT1G68840	RAV2 (REGULATOR OF THE ATPASE OF THE VACUOLAR MEMBRANE)	27.68
AT1G72950	disease resistance protein (TIR-NBS class), putative	27.98
AT2G28400	similar to unknown protein	28.02
AT2G41640	similar to unknown protein	28.34
AT1G72430	auxin-responsive protein-related	28.60
AT3G52450	U-box domain-containing protein	28.71
AT1G56510	disease resistance protein (TIR-NBS-LRR class), putative	28.72
AT3G19580	AZF2 (ARABIDOPSIS ZINC-FINGER PROTEIN 2)	28.96
AT1G17380	JAZ5/TIFY11A (JASMONATE-ZIM-DOMAIN PROTEIN 5)	29.00
AT2G01670	ATNUDT17 (Arabidopsis thaliana Nudix hydrolase homolog 17)	29.47
AT5G63790	ANAC102 (Arabidopsis NAC domain containing protein 102)	30.14
AT1G27730	STZ (SALT TOLERANCE ZINC FINGER)	30.20
AT1G66160	U-box domain-containing protein	30.21
AT4G18950	ankyrin protein kinase, putative	30.72
AT5G63450	CYP94B1 (cytochrome P450, family 94, subfamily B, polypeptide 1)	30.85
AT2G42760	similar to unnamed protein product [Vitis vinifera]	30.94
AT2G38470	WRKY33 (WRKY DNA-binding protein 33); transcription factor	31.65
AT1G76600	similar to unknown protein	31.94
AT4G33050	EDA39 (embryo sac development arrest 39); calmodulin binding	32.48
AT1G20510	OPCL1 (OPC-8:0 COA LIGASE1); 4-coumarate-CoA ligase	33.50
AT4G17500	ATERF-1 (ETHYLENE RESPONSIVE ELEMENT BINDING FACTOR 1)	34.02
AT5G11650	hydrolase, alpha/beta fold family protein	34.06
AT4G08170	inositol 1,3,4-trisphosphate 5/6-kinase family protein	34.12
AT1G27100	similar to unknown protein	34.26
AT5G13220	JAS1/JAZ10/TIFY9 (JASMONATE-ZIM-DOMAIN PROTEIN 10)	34.77
AT2G41410	calmodulin, putative	35.20
AT1G44350	ILL6 (IAA-leucine resistant (ILR)-like gene 6); metallopeptidase	35.55
AT4G24380	hydrolase, acting on ester bonds	36.05
AT1G09940	HEMA2; glutamyl-tRNA reductase	36.53
AT2G01180	ATPAP1 (PHOSPHATIDIC ACID PHOSPHATASE 1)	37.57
AT3G10930	similar to unknown protein [Arabidopsis thaliana] (TAIR:AT5G05300.1)	37.98
AT3G25780	AOC3 (ALLENE OXIDE CYCLASE 3)	38.15
AT1G16370	ATOCT6; carbohydrate transmembrane transporter	38.17
AT5G54170	similar to CP5 [Arabidopsis thaliana] (TAIR:AT1G64720.1)	38.17
AT5G05600	oxidoreductase, 2OG-Fe(II) oxygenase family protein	39.72
AT2G35460	harpin-induced family protein/HIN1 family protein	40.12
AT3G50950	disease resistance protein (CC-NBS-LRR class), putative	40.73
AT3G15500	ATNAC3 (ARABIDOPSIS NAC DOMAIN CONTAINING PROTEIN 55)	40.75
AT5G26030	FC1 (FERROCHELATASE 1); ferrochelatase	40.95

### A relationship between nonadditively expressed *AlloGUTs* and abiotic stress and phytohormone responses

**Figure 4:**
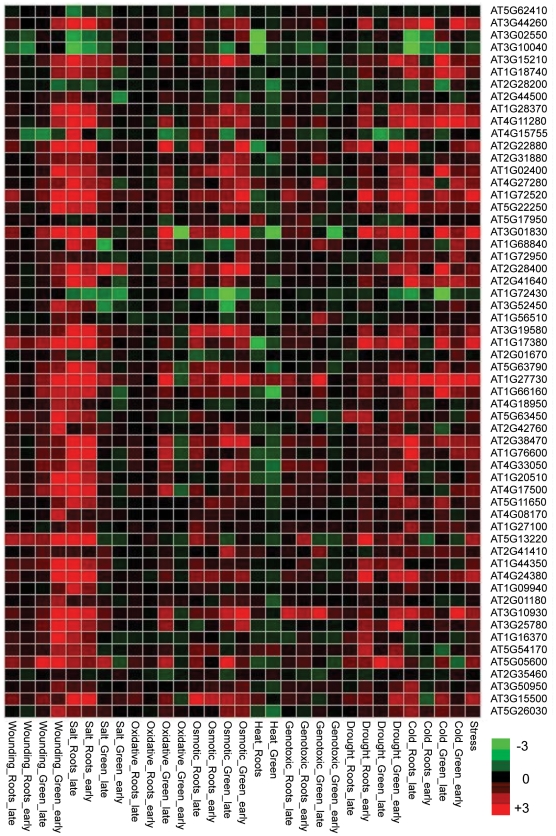
Hierarchical cluster analysis showing the expression levels of the genes induced by stress that matched *AlloGUTs* in allotetraploids. The analysis was carried out using publicly available microarray database (http://www.arabidopsis.org/portals/expression/microarray/ATGenExpress.jsp) and Genevastor. Each row represents log2 expression values of the genes in a given microarray experiment, and each column represents a gene. The color represents relative expression levels of the genes (from −3 green to +3 red).

**Figure 5:**
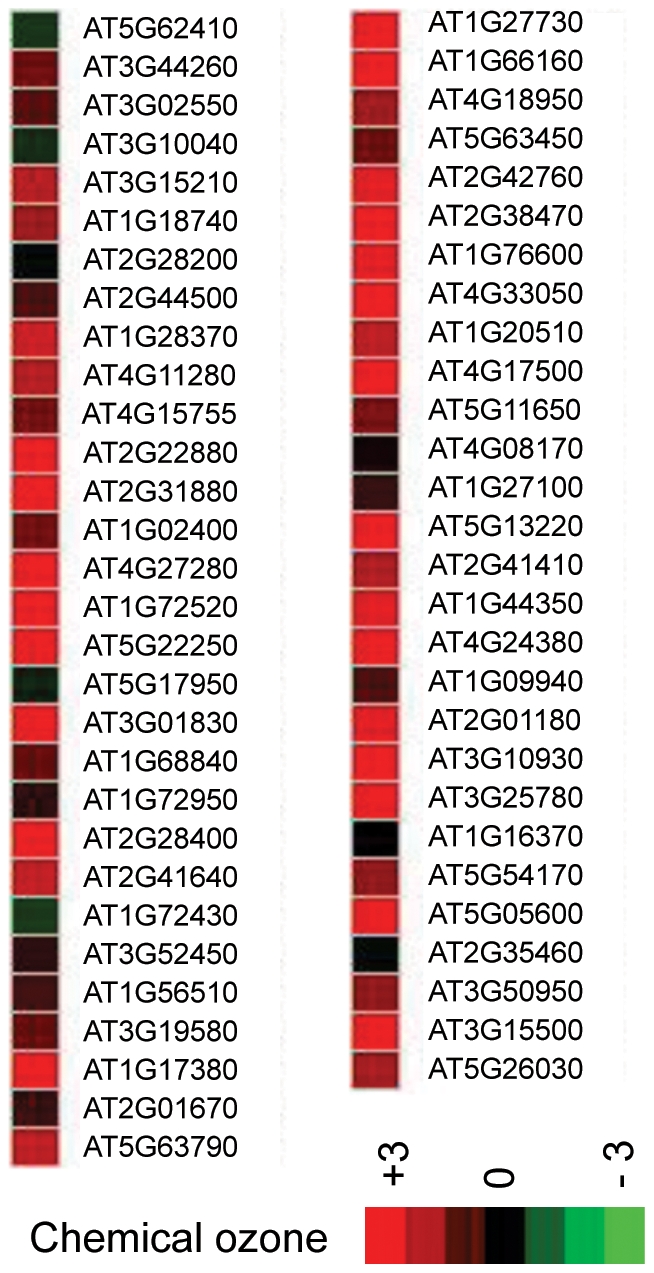
Hierarchical cluster analysis showing the *AlloGUTs* that matched the genes whose expression is induced by ozone stress. The analysis was carried out with publicly available microarray database (http://www.arabidopsis.org/portals/expression/microarray/ATGenExpress.jsp and Genevastor). Each row represents log2 expression values of the genes in a given microarray experiment, and each column represents a gene. The color represents relative expression levels of the genes (from −3 green to +3 red).

To test how nonadditively expressed *AlloGUTs* are affected by physiological and environmental conditions, we performed hierarchical cluster analysis with the Genevastor program [37], [38], using the genes extracted from multiple expression datasets, including global stress [38] and response to hormones and hormone-like chemicals [37]. The comparative data analysis indicated that nonadditively expressed *AlloGUTs* matched the genes whose expression was highly induced by cold, drought, osmotic, oxidative, salt and wounding (Figure 4). In addition, the nonadditively expressed *AlloGUTs* were also highly induced by ozone stress (Figure 5) and tend to alter expression levels in response to the treatments of phytohormones and plant hormone substances, ABA and Methyl Jasmonic acid, cyclohexamide and AgNO_3_ (Figure S2).

Interestingly, under normal growth conditions these stress responsive genes had a fast decay rate, and nonadditively expressed genes were down-regulated in the allotetraploids. This suggests stress-related genes are temporally repressed in allotetraploids under normal conditions and may be inducible under stress conditions.

### Nonadditively expressed *AlloGUTs* also show a nonadditive mRNA decay rate

**Figure 6:**
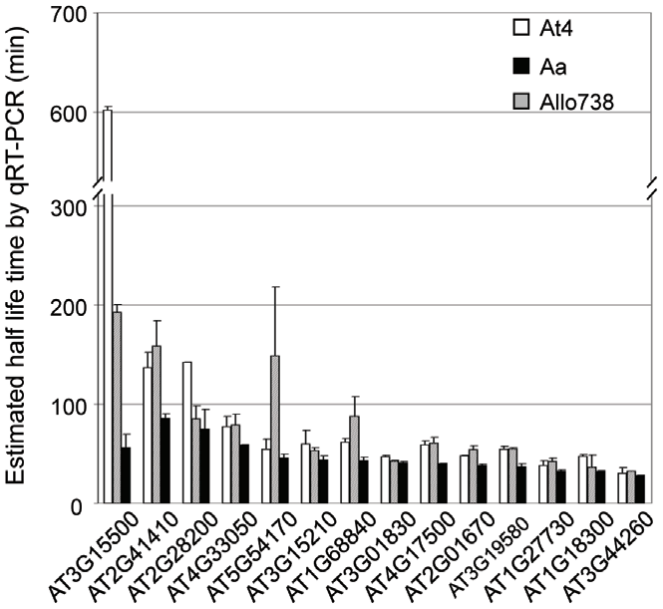
*AlloGUTs* showing more rapid mRNA decay in an allotetraploid (Allo738) than in the progenitors (*A. thaliana* 4x, At4 and *A. arenosa*, Aa). The half-life time was estimated by measuring the decrease rate of *AlloGUT* transcript abundance relative to that of *eIF4A*. Open, gray, and black bars represent values in At4, Aa, and Allo738, respectively.

To test whether nonadditively expressed *AlloGUTs* are subjected to nonadditive mRNA decay in the allotetraploids, we measured the difference in mRNA decay rates between allotetraploids and their progenitors, *A. thaliana* and *A. arenosa*, using qRT-PCR (Figure 6). Among 61 genes tested, 14 (∼25%) of nonadditively expressed *AlloGUTs* were significantly more unstable than the MPV (Figure 6, Table1). The majority of 14 genes tested, except for At3g44260, had a shorter half-life time in the allotetraploid than MPV (Figure 6). According to functional classification by PEDENT, eight genes (∼57%) (At1g27730, At1g68840, At2g28200, At3g15210, At3g15500, At3g19580, At3g44260, and At4g17500) were predicted with a role in transcription. For example, *AtNAC3* (At3g15500) is one of the nonadditively expressed *AlloGUTs*, and its expression is sensitive in response to a wide range of stresses and phytohormones. This transcript had a much shorter half-life in the allotetraploid than in the progenitors. Furthermore, hierarchical cluster analysis indicated that nonadditively expressed *AlloGUTs* are highly correlated with the genes in response to abiotic stress and phytohormones. The data suggest that nonadditive gene expression in the allotetraploids is associated with the nonadditive rate of mRNA decay and stress and phytohormone responses.

### Different decay rates of circadian clock gene transcripts in allotetraploids

**Figure 7:**
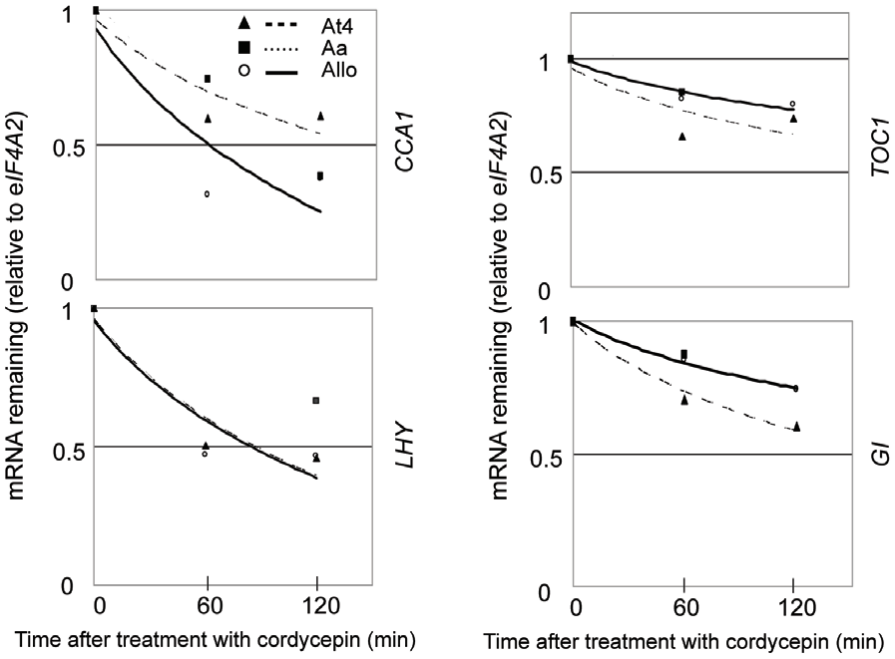
mRNA decay rates of circadian clock genes in allotetraploids relative to their parents. The mRNA decay rates of *CCA1*, *LHY*, *TOC1*, and *GI* were estimated by qRT-PCR. Transcript abundance was shown in At4 (filled triangle), Aa (filled square), and Allo (open circle) with fitted lines of At4 (big dashed line), Aa (thin dashed line), and Allo (solid line).

Nonadditively expressed genes in allotetraploids include those encoding circadian clock regulators, including CIRCADIAN CLOCK ASSOCIATED 1 (CCA1), LATE ELONGATED HYPOCOTYL (LHY), TIMING OF CAB EXPRESSION 1 (TOC1), and GIGANTEA (GI) [14], [39], [40], [41], [42]. The regulation of *CCA1* transcripts by light and its mRNA instability play a role for accurately entraining the oscillator in response to environmental changes [43]. Indeed, we found CCA1 binding motifs and evening elements within 1000-bp upstream region of nonadditively expressed *AlloGUTs.* Among 14 *AlloGUTs*, 7 genes (50%) each contains at least one CCA1 binding site (CBS: AAAAATCT) or evening element [(AA)AATATCT] [39], [44], suggesting that these genes are likely the targets of CCA1 and LHY. We measured the stability of *CCA1*, *LHY*, *TOC1*, and *GI* mRNA in allotetraploid and its progenitors (Figure 7). Transcripts of *CCA1* and *LHY* were relatively unstable, as previously reported [43], whereas the transcripts of their reciprocal regulators, *TOC1* and *GI*, were relatively stable. The decay rate of *CCA1* mRNA in the allotetraploid is relatively faster than MPV, and *LHY* mRNA decreased faster in the allotetraploid than in one of the progenitors. These two nonadditively expressed clock regulator genes also showed a nonadditive rate of mRNA decay.

## Discussion

### Posttranscriptional regulation of cell cycle and stress-related genes in *Arabidopsis* allotetraploids

Transcriptome divergence between allotetraploids and their parents are controlled by transcriptional and/or post-transcriptional regulation [16], [18]. At the transcriptional level, chromatin modifications and DNA methylation mediate activation and silencing of homoeologous genes in allotetraploids or interspecific hybrids [14], [15], [45], [46], [47]. Changes in transcript abundance can also be regulated at the posttranscriptional level. In synthesized wheat allotetraploids, activation of retrotransposon affects the expression of neighboring genes [48]. RNA-mediated gene expression may be associated with the equilibrium between mRNA accumulation and stability during growth, development, and physiological transitions [49], [50], [51], [52], [53]. Here we provided the first evidence for mRNA instability study in nonadditive gene expression in *Arabidopsis* allotetraploids. Our data indicated that the genes with unstable transcripts are correlated with nonadditive levels of steady mRNAs in the allotetraploids. Therefore, a proportion of nonadditively expressed genes in the allotetraploids is caused by unstable transcripts.

The relatively small number of *alloGUTs* identified in the study can be due to several factors. One possibility is that unstable mRNAs have low steady state levels, which may not be detectable by microarrays. For example, circadian clock genes such as *CCA1* and *LHY* have unstable transcripts [43] but were not identified by microarrays. Alternatively, cross-hybridization of the probes between species could be another factor that have under-estimated the number of genes with unstable transcripts. One might expect that additional *GUTs* will be identified using high-resolution next-generation sequencing technology (RNA-seq) [54]. Alternatively, the noise associated with quantification of mRNA stability may also account for some differences observed.

GOSlim classifications show that like *AtGUTs*, *AlloGUTs* are found in all plant cellular processes. The proportion of *AlloGUTs* is enriched 2-fold or more than that of all genes in several GO groups, including transcription, cell cycle and DNA processing, cellular communication, and biogenesis of cellular components. The majority of these genes are involved in cell cycle regulation and DNA metabolism, a process that requires rapid and cyclic regulation. For example, in yeast, cell cycle and mating-type genes have unstable transcripts [22], [24], [55], [56]. Significantly, *AlloGUTs* were enriched 3-fold more transcripts that are related to exogenous stimulus such as cell rescue, defense and virulence and interaction with the environments (Figure 2), suggesting that these genes are regulated by the rate of mRNA decay. Rapid decay of mRNA is a mechanism for fast response to environmental and external signals such as biotic and abiotic stresses [30], [31]. The data are consistent with high retention rates of duplicate genes and high expression divergence between duplicate genes than single-copy genes in the process of polyploidization [36], [57]. Nonadditive expression of stress-related genes is also overrepresented in other allopolyploids such as wheat [58], [59] and cotton [60], [61], [62]. This may also suggest that *AlloGUTs* are involved in these processes to facilitate faster mRNA adjustment in allotetraploids and hybrids than in diploids [21], [24], [30], [35]. During evolution, rapid mRNA turnover of the transcripts made from additional genes in allopolyploids is likely to facilitate selection and adaptation for environmental niches [6].

### *AlloGUTs* are associated with nonadditive gene expression in allotetraploids in response to stresses and phytohormones

A significant finding is that ∼ 30% of *AlloGUT*s overlap with the nonadditively expressed genes in allotetraploids [14] (Figure 3A). Moreover, there is a statistically significant negative correlation between the expression levels of *AlloGUTs* and nonadditively expressed genes in the allotetraploids. The unstable mRNA transcripts tend to be nonadditively repressed in allopolyploids, suggesting a role of mRNA instability in nonadditive gene expression in allopolyploids. In other words, different transcriptome profiles of nonadditively regulated genes can be generated by different sensitivities of the mRNA degradation rate during allopolyploidization.

The *AlloGUTs* that overlap with nonadditively expressed genes are enriched 2–7-fold in GOSlim groups in response to stress, abiotic or biotic stimulus, signal transduction and transcription. There is also a 1.5-fold enrichment of the GO groups in transcription, developmental processes, other cellular processes and other metabolic processes. Compared to additively expressed *AlloGUTs*, nonadditively expressed *AlloGUTs* are significantly correlated with the genes in GOslim groups in response to cold, drought, osmotic, salt, and wounding stress. In response to environmental stimuli, an increase or decrease in mRNA abundance might be attained either by adjusting the rate of transcription or the rate of degradation, as demonstrated in many studies [25]. In addition, nonadditively expressed *AlloGUTs* are correlated with the genes induced by Methyl jasmonic acid and plant hormone substances, such as AgNo3, cycloheximide, and 2,3,5-triiodobenzoic acid. In cotton allopolyploids, expression ratios of *AdhA* homeologs vary considerably during seedling and fruit development [62]. Homeologous gene expression is altered by abiotic stress treatments, including cold, dark, and water submersion. These data collectively suggest that nonadditive gene expression and unstable transcripts provide a general mechanism in response to external signals such as biotic and abiotic stress as well as to internal signals such as phytohormones and intergenomic interactions.

Nonadditively expressed *AlloGUTs* also show nonadditive rate of mRNA decay in both parents. Among them, 25% of *AlloGUTs* have a faster decay rate in the allotetraploid than in the parents, 57% of which encode transcription factors. One gene, *AtNAC3* (At3g15500), encodes one member of the NAC transcription factor family. The expression of *AtNAC3* is highly induced by drought, high salinity, and abscisic acid [63]. Genome-wide analysis of *AtNAC3*-overexpressing transgenic plants showed a significant increase in drought tolerance, which was correlated with upregulation of several stress-inducible genes. The transgenic plants overexpressing *AtNAC3* also show significantly higher survival rate (100%) under drought stress [63]. Another gene, *AtERF1* (At4g1750), encodes EHTYLENE RESPONSE FACTOR1, a transcription factor, which controls the expression of pathogen responsive genes and prevents disease progression. Overexpressing *AtERF1* in the transgenic plants increases resistance to pathogens [64], as well as complements defense response defects of *coronative insensitive 1* (*coi1*) and *ethylene insentive2* (*ein2*) mutants [65]. Interestingly, the transgenic plants overexpressing *AtERF1* are significantly smaller than wild-type plants.

The above data are consistent with the observation that many stress responsive genes are generally repressed in the allotetraploids under normal conditions [14]. Allotetraploids might have developed a mechanism that requires low maintenance at the transcript level for these genes in response to environmental changes. Under stress conditions, adjusting the ratio of unstable and stable mRNA transcripts may help rapidly facilitate the proper mRNA levels in response to stress. Whether or not the regulation of stress responsive genes is correlated with increased growth vigor in allopolyploids remains to be investigated.

### Circadian clock genes produce unstable transcripts in allotetraploids and their progenitors

Plants are sessile and need to prepare for and respond to all environmental changes including biotic and abiotic stresses. Recent studies suggested that 10–90% genes in *Arabidopsis* are governed by circadian clock regulation [40], [44], [66], [67]. CCA1 and LHY are members of a small family of single Myb domain transcription factors and have redundant but non-overlapping functions. CCA1 and LHY bind to the promoter of *TOC1* and downstream genes in photosynthesis and starch metabolism [39], [44]. *CCA1* expression is negatively regulated by TOC1 and CHE [42], [68]. Accurate entrainment of circadian rhythms is controlled by a combination of *CCA1* transcripts level and mRNA degradation by light [43]. We found that *CCA1* and *LHY* transcripts are unstable and show a relatively high rate of mRNA turnover. Compared to *CCA1* and *LHY*, *TOC1* and *GI* transcripts are relatively stable. Interestingly, these clock genes are nonadditively expressed in allotetraploids [14], [39], and their expression levels are negatively correlated with their decay rates (this study). Specifically, *CCA1* and *LHY* mRNA has a relatively lower level of stability in the allotetraploid than in both parents. The data suggest in addition to transcriptional regulation, some circadian clock genes such as *CCA1* and *LHY* are regulated through mRNA decay at the posttranscriptional level. Together, the available data indicate that transcriptional and posttranscriptional regulation of circadian clock genes, stress responsive genes, and many other *GUTs* provides a general mechanism for rapid changes in gene expression induced by “genome shock” [69] in response to interspecific hybridization and allopolyploidization. Posttranscriptional regulation of mRNA transcripts offers a unique mechanism to discriminate the transcripts between homoeologous loci in the allopolyploids. It will be interesting to investigate how these homoeologous transcripts are discriminated and degraded.

### Conclusions

The current data support a role of mRNA instability in nonadditive expression of genes in *Arabidopsis* allopolyploids. We found ∼1% of genes with allotetraploids unstable transcripts (*AlloGUTs*) in *Arabidopsis* and a half-life time of less than 60 minutes, ∼30% of which matched the nonaddtively expressed genes that are repressed in the allotetraploids. All *AlloGUTs* and nonadditively repressed *AlloGUTs* are significantly enriched with GOSlim classifications of response to stress, environment, and phytohormones. These *AlloGUTs* overlap with the genes that are highly induced by abiotic stress, including cold, drought, osmotic, salt, and wounding, and plant hormones and substances, including Methyl jasmonic acid and cycloheximide. Expression of circadian clock genes such as *CCA1* and *LHY* is controlled by transcriptional regulation as well as posttranscriptional regulation through mRNA decay. The data collectively suggest that transcriptional and posttranscriptional regulation affects expression of homoeologous genes related to stress response, light signaling, and hormone interactions in allopolyploids, which may provide a flexible and rapid response to external and internal stimuli as a consequence of polyploidization.

## Materials and Methods

### Plant Material and RNA sample preparation for half-life time calculation

All plants were grown under sterile conditions on plates containing 1x Murashige and Skoog salts, 1x Gamborg's vitamins and 1% sucrose for 2 weeks at 22°C. Light regime was 16-hr light and 8-hr dark in a growth chamber. Plant materials included *A. thaliana* autotetraploid (At4, accession no. CS3900, 2*n*  =  4*x*  = 20), tetraploid *A. arenosa* (Aa, accession no. CS3901, 2*n*  =  4*x*  = 32), and resynthesized *A. suecica* allotetraploid lines in F_8_ generation (Allo733 and 738, 2*n*  =  4*x*  = 26, accession no. CS3895-3896) [13], [14], [15]. Two-week old seedlings were used for all analyses.

The plants were incubated in a beaker containing incubation buffer for 30 min [32]. After the incubation, 3′-decoyadenosine (cordycepin) was added to a final concentration of 200 mg/ml for all seedlings. Cordycepin was used as a transcription inhibitor because it works better than other inhibitors such as Actinomycin D and it is more effectively to penetrate into leaf tissues [30], [32], [70]. Treated leaves were harvested at each time point, 0, 60, and 120 min, after inhibitor treatment, and immediately frozen in liquid nitrogen. Total RNA was isolated using Plant RNA reagent (Invitrogen, Carlsbad, CA). Each RNA sample was quantified by measuring the 260/280 ratio using Nano drop and by agarose-formaldehyde gel electrophoresis. Quantified total RNA was subjected to mRNA isolation by Micro-fast Track 2.0 mRNA isolation kit (Invitrogen). Isolated mRNA was reverse-transcribed to synthesize cDNA using Cy3- or Cy5- labelled dCTPs (Amersham Biosciences, Piscataway, NJ) that was used as probes for microarray analysis, as previously described [14].

### Quantitative RT-PCR

Approximately 5 mg of total RNA treated with DNase Ι was reverse-transcribed using SuperScriptII (Invitrogen), following the manufacturer's recommendation. The synthesized cDNA was diluted 1∶5 in DEPC water and subjected to quantitative RT-PCR (qRT-PCR) analysis using SYBR Green Supermix (ABI Biosystems, Foster City, CA) in an ABI 7500 instrument (ABI Biosystems). The qRT-PCR conditions were optimized to maximize the amplification efficiency as previously described [71]. In this study, the gene *eIF4A2* was used as an internal control to estimate the relative transcript level of the gene tested. The primers were provided in Table S3.

### Spotted oligo-gene microarrays and data analysis

Spotted oligo-gene microarrays with 26,090 annotated genes were used for monitoring mRNA stability in allotetraploids according to the published protocol [14], [71], [72]. A total of 16 slides were used for two-time course comparisons (0 min vs. 120 min), including two biological and two technical replications. Each biological replicate consists of two dye-swaps (four slides) that were hybridized with reverse-labeled probes using one mRNA sample from the allotetraploid and another mRNA sample from a mix of RNAs from two parents (mid-parent value, MPV) (Figure 1A). For microarray hybridization, 500 ng of mRNA were used to synthesize cDNA in each labelling reaction using Cy3- or Cy5-dCTP (Amersham Biosciences, Piscataway, NJ). Probe labelling, slide hybridization, and washing were according to a published protocol [72]. After the slides were scanned using Genepix 4000B, raw data were collected using Genepix Pro4.1. The replicated data from the dye-swap of each comparison were extracted and normalized using Acuity 4.0 software (Molecular Devices, http://www.moleculardevices.com). Quantified values in each dataset were analyzed by t-test using a statistical significance level of α = 0.001. The common genes between the two datasets with reproducibly normalized intensity ratios of ≥2 were used for further analysis. The difference of the transcript abundance between each time point can be used to calculate the half-life by the equation ln (Normalized Ratio)  =  -*k*_decay_, with *t_1/2_*  = 0.693/*k*_decay_, since mRNA degradation generally obeys first-order kinetics, which is common in fast mRNA degradation [30], [73]. Gene Ontology (GOSlim) of *AlloGUTs* was classified using TAIR release (November 2009) (ftp://ftp.arabidopsis.org/home/tair/Ontologies/Gene_Ontology/ATH_GO_GOSLIM.txt) and PEDANT (http://mips.gsf.de/proj/thal/db/index.html). Clustering analysis of gene expression was performed using CLUSTER and TREEWIEW [74] (http://rana.lbl.gov/EisenSoftware.htm). Our microarray data were comparatively analyzed with the published data [14] to determine if nonadditively expressed *AlloGUTs* were enriched in GOSlim groups in response to stress [38], hormones [37], and circadian regulation [75]. The data were downloaded from http://www.arabidopsis.org/portals/expression/microarray/ATGenExpress.jsp. Visual display of expression profiles from multiple experiments was performed in Genevestigator [76] (https://www.genevestigator.com/gv/index.jsp).

All microarray data are MIAME compliant http://www.ncbi.nlm.nih.gov/geo/info/MIAME.html and deposited in GEO http://www.ncbi.nlm.nih.gov/geo/with the accession GSE26065.

## Supporting Information

Figure S1 **The percentages of the genes in GOSlim category that are nonadditively expressed** ***AlloGUTs*** **(61 genes), compared to all** ***AlloGUTs*** **.** The ratios in the y-axis were calculated using the observed percentage of the nonadditively expressed *AlloGUTs* divided by the expected percentage of all annotated genes in the *Arabidopsis* genome. The dashed line shows the observed percentage of the genes that are *AlloGUTs* in a microarray experiment equal to that of the expected genes in the whole genome (100%).
(TIF)

Figure S2 **Hierarchical cluster analysis showing** ***AlloGUTs*** **that matched the genes whose expression is induced by hormone and chemical treatments.** The analysis was carried out with publicly available microarray database (http://www.arabidopsis.org/portals/expression/microarray/ATGenExpress.jsp and Genevastor). Each row represents a microarray experiment and each column represents a gene. The color represents the relative expression level of each experimental group (Red, up and green, down).
(TIF)

Table S1 ***Arabidopsis*** **allotetraploid genes with unstable transcripts (** ***AlloGUTs*** **).**
(XLS)

Table S2 **Statistic tests for significance between microarray datasets.**
(DOC)

Table S3 **Primer sequences used in the analysis.**
(DOC)
