# Supplementary figures and images for: Unstable Transcripts in Arabidopsis Allotetraploids Are Associated with Nonadditive Gene Expression in Response to Abiotic and Biotic Stresses
Source: PLoS One. 2011 Aug 29;6(8):e24251. doi: 10.1371/journal.pone.0024251 (PMC3163679; doi:10.1371/journal.pone.0024251)

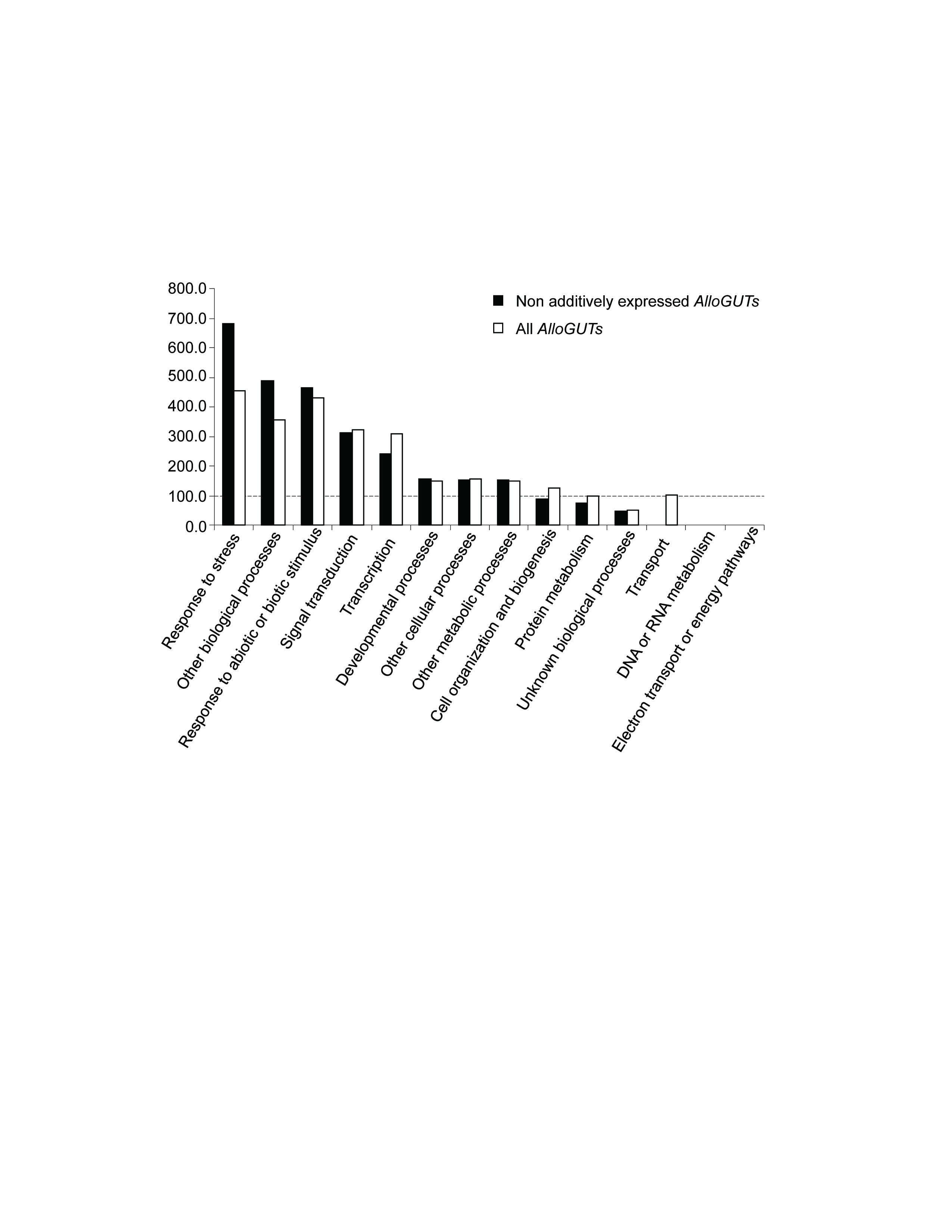

Supplement: Figure S1 — The percentages of the genes in GOSlim category that are nonadditively expressed AlloGUTs (61 genes), compared to all AlloGUTs . The ratios in the y-axis were calculated using the observed percentage of the nonadditively expressed AlloGUTs divided by the expected percentage of all annotated genes in the Arabidopsis genome. The dashed line shows the observed percentage of the genes that are AlloGUTs in a microarray experiment equal to that of the expected genes in the whole genome (100%). (TIF) [file pone.0024251.s001.tif]

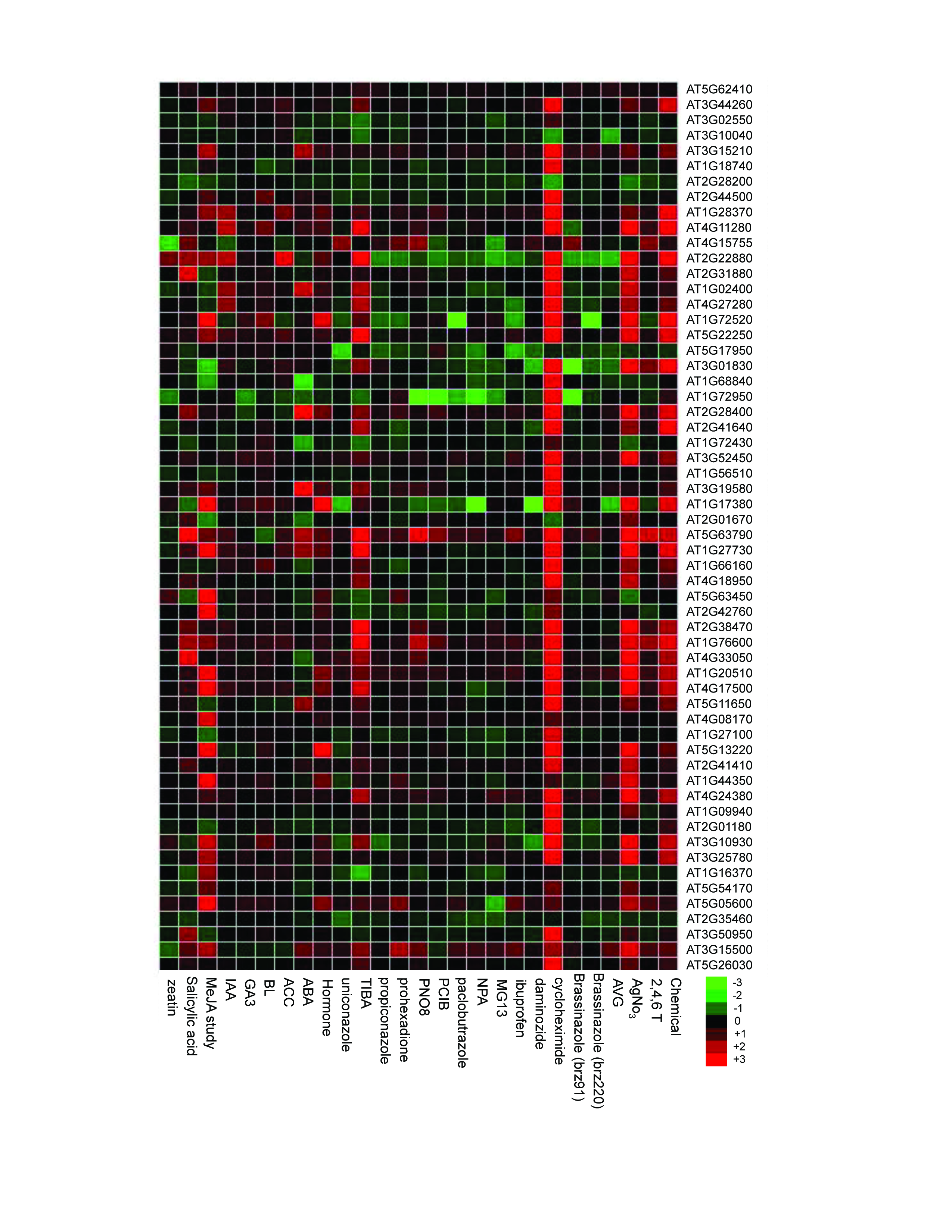

Supplement: Figure S2 — Hierarchical cluster analysis showing AlloGUTs that matched the genes whose expression is induced by hormone and chemical treatments. The analysis was carried out with publicly available microarray database (http://www.arabidopsis.org/portals/expression/microarray/ATGenExpress.jsp and Genevastor). Each row represents a microarray experiment and each column represents a gene. The color represents the relative expression level of each experimental group (Red, up and green, down). (TIF) [file pone.0024251.s002.tif]
