# Supplementary material for: Unstable Transcripts in Arabidopsis Allotetraploids Are Associated with Nonadditive Gene Expression in Response to Abiotic and Biotic Stresses
Source: PLoS One. 2011 Aug 29;6(8):e24251. doi: 10.1371/journal.pone.0024251 (PMC3163679; doi:10.1371/journal.pone.0024251)
Supplement: Table S2 — Statistic tests for significance between microarray datasets. (DOC) [file pone.0024251.s004.doc]

**Table S2. Statistic tests for significance between microarray datasets.**

|  | Non-additive | Additive | Total |
| --- | --- | --- | --- |
| Unstable transcripts (t1/2<60min ) | 61 | 147 | 208 |
| Stable transcripts (t1/2<60min) | 744 | 24048 | 24792 |
| Total | 805 | 24195 | 25000 |

Chi square test for statistical significance

Degrees of freedom: 1

Chi-square = 460.945960772186

*p* is less than or equal to 0.001. Therefore this distribution is significant.
