# Supplementary material for: Unstable Transcripts in Arabidopsis Allotetraploids Are Associated with Nonadditive Gene Expression in Response to Abiotic and Biotic Stresses
Source: PLoS One. 2011 Aug 29;6(8):e24251. doi: 10.1371/journal.pone.0024251 (PMC3163679; doi:10.1371/journal.pone.0024251)
Supplement: Table S3 — Primer sequences used in the analysis. (DOC) [file pone.0024251.s005.doc]

**Table S3. Primer sequences used in the analysis**

| **(A)** Primers for SSCP analysis | |
| --- | --- |
| **Name** | **Sequences** |
| AT3G15500 F | TGGTTCGTCGACATCTTCTTCG |
| AT3G15500 R | CGGGTCAACACTAAAACCCTGA |
| A1G54270 F | TTCCAGCTTCTCCCACCAAAGA |
| AT1G54270 R | GAGGGAAGCTCCTCAACAACCA |
| **(B)** Primers for qRT-PCR | |
| **Name** | **Sequences** |
| AT3G15500 F | CGAGTGGTCGTGAATACAGCAA |
| AT3G15500 R | GAGACGAAGAAGATGTCGACGAA |
| AT2G28200 F | CGTGTACTTTGTTTGCCTTGTTG |
| AT2G28200 R | CCCTAGCTTCACAGGCAAGTAAC |
| AT4G33050 F | ACACCTTTTGTAACGTAGTTGCAGC |
| AT4G33050 R | CTAGACCTGTCACCGAGCTTGAT |
| AT5G54170 F | AACGCAGGAGGAACAAAAGC |
| AT5G54170 R | TGTTGCGGTTGTCTGCAGTT |
| AT3G15210 F | GTTATCAGATCCCGATGTCGC |
| AT3G15210 R | TCCCCATCAGGTCCAAAAAGTA |
| AT1G68840 F | TTCAACGAGCAAGAAGAAGCTG |
| AT1G68840 R | GGAACACCGTAACGCAAACATA |
| AT3G01830 F | TCGTTGGAGCTCGAGGACTT |
| AT3G01830 R | TGCCTCCTCTCCTTCTTCCAC |
| AT4G17500 F | GAGGAGTGAGACAAAGGCCG |
| AT4G17500 R | GGTCTCTAATCTCCGCCGC |
| AT2G01670 F | AATGCTTCTTTCATCCACCAATC |
| AT2G01670 R | TGTAACCGAAGCAAGAGAAGCTT |
| AT3G19580 F | TTTCTTGATGACTTGGTCGTGATC |
| AT3G19580 R | TGACCGGTAAAAAACCGCTTT |
| AT1G27730 F | GAGAAGCATGAGGCAAAAAGCTA |
| AT1G27730 R | CACCAAAACCTCACTGAGGAAGA |
| AT3G44260 F | TTCACATCATACACACGCTCCC |
| AT3G44260 R | GTTTAAGCGCGTGATGAGAGTG |
| AT5G26030 F | GACGATCACGAATCGTGGATT |
| AT5G26030 R | GCTTGCTCAAACACATTCGTCT |
| AT4G11280 F | AACCGAACTATGGCGTGTGATT |
| AT4G11280 R | GGAGACACGTTGAGCTTCACTTG |
| At2G46830 F | CCTCGTCAGACACAGACTTCCA |
| AT2G46830 R | CCGCAGTAGAATCAGCTCCAATA |
| AT1G01060 F | GGGACAAAGACTGCTGTTCAGAT |
| AT1G01060 R | TTTGTGAAGAACTTTTGTGCATGA |
| AT5G61380 F | GTTGATGGATCGGGTTTCTC |
| AT5G61380 R | TCATGACCCCATGCATACAG |
| AT1G22770 F | TCGAGCAACTTCATCATCACAAA |
| AT1G22770 R | GCTAATGGAGCTGGTGTCATACTG |
| AT1G54270 F | GACCTCCCAACTCAGCCAGAG |
| AT1G54270 R | TCCACTTCTTCCGATACGGTG |
